# Identification of DVA Interneuron Regulatory Sequences in *Caenorhabditis elegans*

**DOI:** 10.1371/journal.pone.0054971

**Published:** 2013-01-28

**Authors:** Carmie Puckett Robinson, Erich M. Schwarz, Paul W. Sternberg

**Affiliations:** 1 Division of Biology and Howard Hughes Medical Institute, California Institute of Technology, Pasadena, California, United States of America; 2 Department of Neurology and VA Greater Los Angeles Healthcare System, Keck School of Medicine, University of Southern California, Los Angeles, California, United States of America; The University of Chicago, United States of America

## Abstract

**Background:**

The identity of each neuron is determined by the expression of a distinct group of genes comprising its terminal gene battery. The regulatory sequences that control the expression of such terminal gene batteries in individual neurons is largely unknown. The existence of a complete genome sequence for *C. elegans* and draft genomes of other nematodes let us use comparative genomics to identify regulatory sequences directing expression in the DVA interneuron.

**Methodology/Principal Findings:**

Using phylogenetic comparisons of multiple *Caenorhabditis* species, we identified conserved non-coding sequences in 3 of 10 genes (*fax-1*, *nmr-1*, and *twk-16)* that direct expression of reporter transgenes in DVA and other neurons. The conserved region and flanking sequences in an 85-bp intronic region of the *twk-16* gene directs highly restricted expression in DVA. Mutagenesis of this 85 bp region shows that it has at least four regions. The central 53 bp region contains a 29 bp region that represses expression and a 24 bp region that drives broad neuronal expression. Two short flanking regions restrict expression of the *twk-16* gene to DVA. A shared GA-rich motif was identified in three of these genes but had opposite effects on expression when mutated in the *nmr-1* and *twk-16* DVA regulatory elements.

**Conclusions/Significance:**

We identified by multi-species conservation regulatory regions within three genes that direct expression in the DVA neuron. We identified four contiguous regions of sequence of the *twk-16* gene enhancer with positive and negative effects on expression, which combined to restrict expression to the DVA neuron. For this neuron a single binding site may thus not achieve sufficient specificity for cell specific expression. One of the positive elements, an 8-bp sequence required for expression was identified in silico by sequence comparisons of seven nematode species, demonstrating the potential resolution of expanded multi-species phylogenetic comparisons.

## Introduction

Neurons express a largely overlapping set of genes required for their general function as a neuron. The specific identity of each individual neuron, in turn, requires the expression of distinct sets of genes comprising terminal gene batteries [1], [2], [3]. In a few neurons, the regulatory sequences determining the expression of sets of genes comprising the terminal gene battery have been identified, but most remain obscure. Identifying these regulatory sequences remains a challenging problem due to the complexity of the nervous system [3].

**Figure 1:**
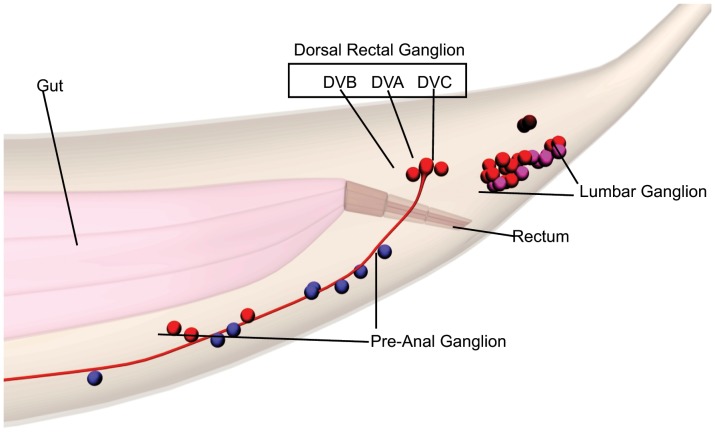
Schematic images of *C. elegans* tail ganglion and dorsal rectal ganglion neurons. The labeled ganglion are the Pre-anal ganglion (PA), Lumbar ganglion (LG) and Dorsal Rectal Ganglion (DRG). The individual neurons comprising the DRG are DVB, DVA and DVC in the black box. The gut is in pink and the rectum shown in darker brown. The images were derived from www.WormBase.org, by Christopher Grove (Caltech).

The *C*. *elegans* hermaphrodite nervous system is relatively simple, with defined cell lineages and anatomy [4]. The ability to identify neurons by Nomarski optics and to examine cell-specific gene expression by transgenic reporters makes *C. elegans* useful to investigate pertinent regulatory sequences. A few *C*. *elegans* neurons are unpaired, including DVA, an interneuron in the tail required for the worm to integrate mechanosensory information and to sense how its own body bends as it moves [5], [6]. The DVA neuron is located in the dorsal rectal ganglia (DRG) between DVB and DVC; each of these three neurons has distinct functions and lineal origins (Figure 1).

Identification of regulatory elements by deletion analysis is unbiased, but laborious. Phylogenetic footprinting has also been used as a shortcut to regulatory motifs [7], [8]. These approaches in *C*. *elegans* have identified enhancer motifs that direct expression broadly in neurons, in classes of neurons and selectively in individual neurons [9], [10], [11] [12]. Shared motifs binding the transcription factors (TF’s) AST-1 and UNC-3 have been identified for the co-regulation of genes required, respectively, for the expression of dopaminergic or cholinergic neurotransmitter phenotypes [11], [13]. These studies lead to the hypothesis that neuron-specific sequence motifs constitute a simple combinatorial code regulating terminal gene expression. For example, deletion analysis of eight genes expressed in the interneuron AIY identified a 16-bp motif regulated by the cooperative binding of CEH-10 and TTX-3 [10]. Similar analyses identified a 12-bp ASE motif bound by CHE-1 [14] and a bipartite A/T rich core consensus sequence was identified in the regulatory regions of chemoreceptor genes expressed in AWB. In contrast to the regulatory motif found in AIY neurons, the AWB motif was not conserved in *C. briggsae* [15].

There are 941 transcription factors in *C*. *elegans* [16], [17], [18] potentially available for the cis-regulation of only 302 neurons in hermaphrodites [4]. The studies of AIY, ASE and AWB are consistent with the model that *C*. *elegans* utilizes neuron-specific regulatory codes for the regulation of the terminal gene battery [10]. A second model would be that neuron-specific gene expression relies on complex modular combinations of positive or negative elements [19]. A third possibility is that both neuron-specific and complex modular elements regulate the terminal gene battery of each neuron. In the latter two models, a broad analysis of regulatory motifs in a terminal gene battery would not usually identify neuron-specific motifs regulating that terminal gene battery.

Here we use comparative genomics to analyze genes expressed in the DVA interneuron of *C. elegans*. By combining newly sequenced nematode species and phylogenetic footprinting [8], [20] we attempted to reduce the experimental work necessary for the identification of regulatory regions. We applied this method to genes identified as being expressed in DVA, but with wider neuronal expression, along with a mutational analysis of the previously described *twk-16* enhancer that shows highly restricted expression in DVA [21].

## Results

### Phylogenetic Footprinting of DVA-expressed Genes

**Figure 2:**
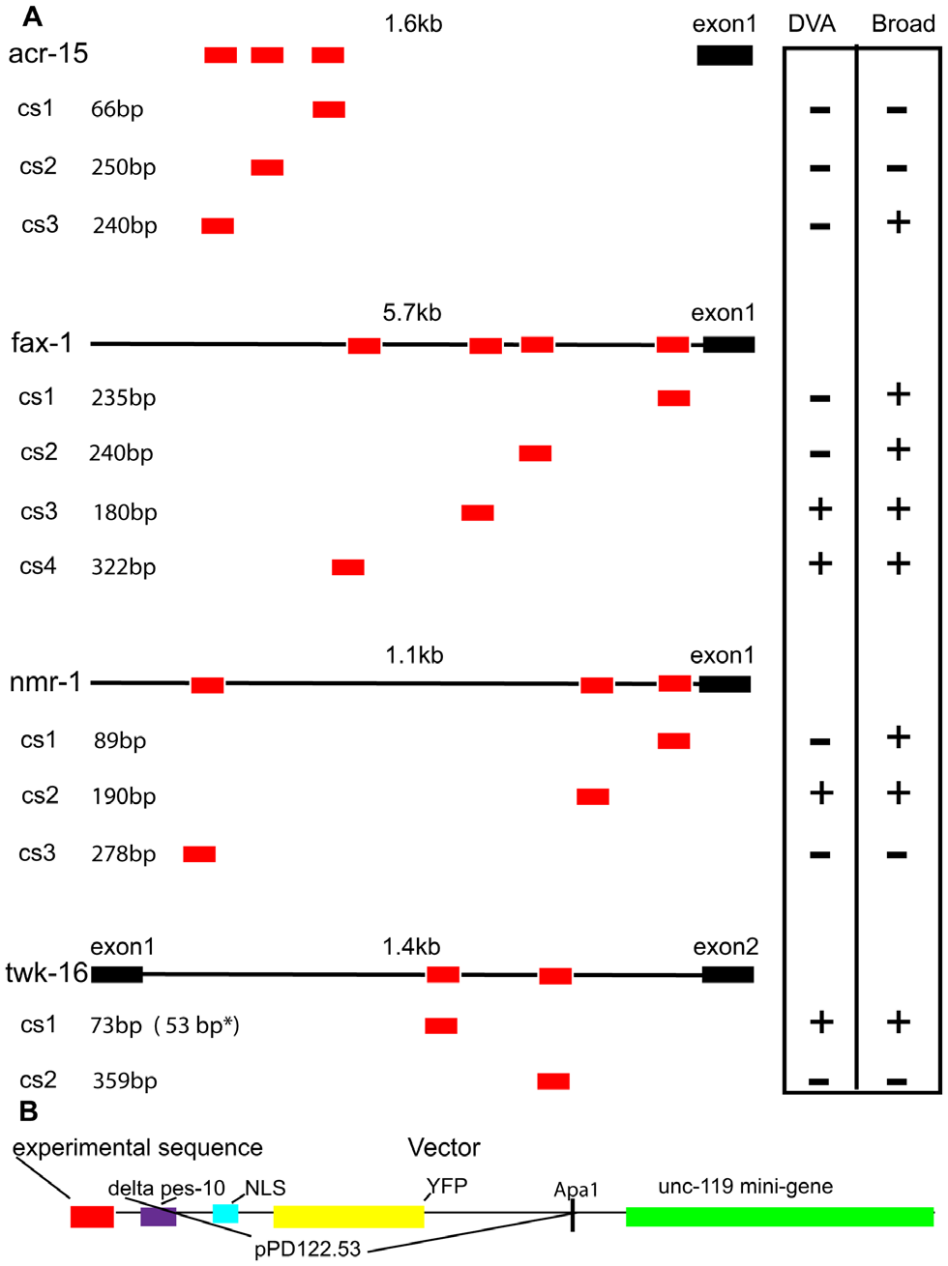
A. Conserved regions analyzed for DVA expression. Relative location of conserved regions identified by MUSSA from the *acr-15*, *fax-1*, *nmr-1* and *twk-16* genes. Conserved regions are depicted as red boxes below the corresponding gene and denoted as cs1-cs4 based on their relative position from the first exon in black. The intergenic regions are shown as a black line with the size (kb) above. Neuronal expression is shown in the vertical oriented box as+or - under DVA or Broad. The parentheses and asterisk (53 bp*) following the 73 bp twk-16 cs1 region denotes that the 53 bp fragment of the 73 bp cs1 region expressed in DVA and Broadly. The 73 bp twk-16 cs1 region does not show expression. **B. Expression vector**. The features of the PCR expression vector are denoted by colors: experimental sequences (red); Δpes-10 (purple); nuclear localization signal (NLS) (blue); YFP (yellow); and derived from the Fire Vector pPD122.53. The *unc-119* promoter and *unc-119* mini-gene are in green. Experimental sequences were fused by PCR to this expression vector to form a single PCR product.

We looked for conserved, ungapped DNA sequences of 10 genes expressed in DVA [22]. Specifically, we used MUSSA [7], [8] for a three-way comparison of *C*. *elegans*, *C*. *briggsae* and *C*. *remanei* orthologs of: *acr-15* (acetylcholine receptor subunit), *fax-1* (nuclear receptor gene), *glr-4* and *glr-5* (glutamate receptor subunits), *nmr-1* (NMDA receptor subunit), *ser-2* (tyramine receptor), *ser-4* (metabotropic serotonin receptor), *trp-4* (transient receptor potential channel), *twk-16* (TWK potassium channel) and *zig-5* (secreted immunoglobulin superfamily protein). Genes with ≤4 conserved regions were selected for experimental analysis to reduce the number of transgenic experiments necessary. This criterion excluded *glr-4*, *glr-5*, *ser-2*, *ser-4*, *trp-4* and *zig-5*. Conserved sequences were named in order of their proximity to the first exon of each gene as.cs1,.cs2,.cs3, respectively (Figure 2A). The conserved regions of *acr-15* [23] (3 regions), *fax-1* [24] (4 regions), and *nmr-1* [25] (3 regions; Figure S1) were fused to a reporter cassette (Figure 2B) and examined in transgenic lines.

**Figure 3:**
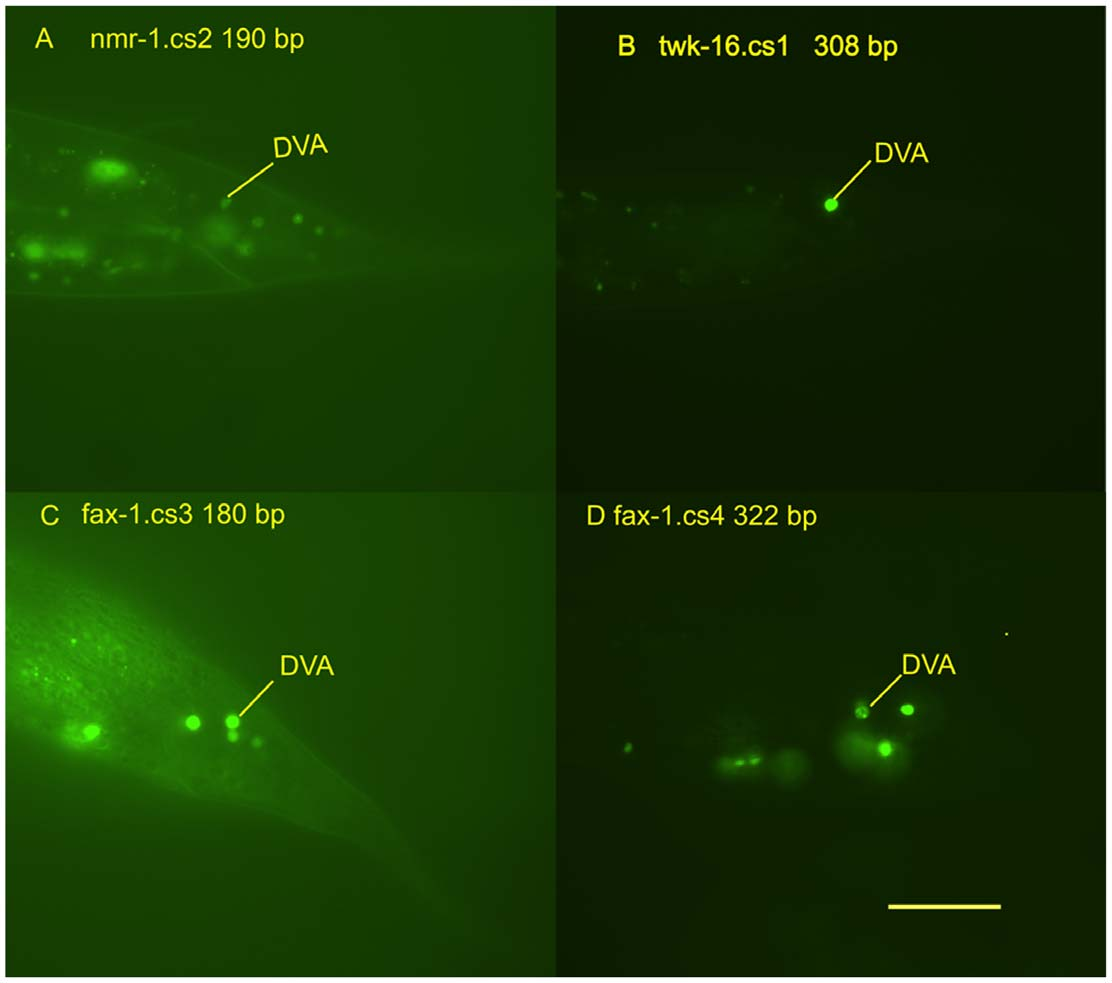
Conserved regions driving expression in DVA. Panels **A–D** are photomicrographs of the tail region of transgenic L4-adult *C. elegans*. DVA expression is denoted by a yellow line identifying the DVA neuron. The gene name is followed by the conserved region numbered by its position relative to the first exon. **A**. DVA expression of the 190 bp conserved region 2 of *nmr-1* (nmr-1.cs2). **B**. DVA expression of the 308 bp fragment containing conserved region 1 of *twk-16* (twk-16.cs1). **C**. DVA expression of the 180 bp conserved region 3 of *fax-1* (fax-1.cs3). **D**. DVA expression of the 322 bp conserved region 4 of *fax-1* (fax-1.cs4). Scale bar = 20 µm.

This analysis identified four conserved regions that directed expression in DVA: a 308 bp fragment containing the conserved region (twk-16.cs1) previously identified by Salkoff [21] a 190-bp conserved region (nmr-1.cs2); a 180 bp conserved region (fax-1.cs3) and a 322-bp conserved region (fax-1.cs4; Figure 2A). Examples of the expression seen in DVA and other tail neurons with these regions are shown in Figure 3. The conserved regions of the *nmr-1* and *fax-1* intergenic regions produced broader neuronal expression in the head and ventral cord in contrast to the restricted expression seen with the *twk-16* intronic region.

### Phylogenetic Comparisons of *twk-16* Genes

**Figure 4:**
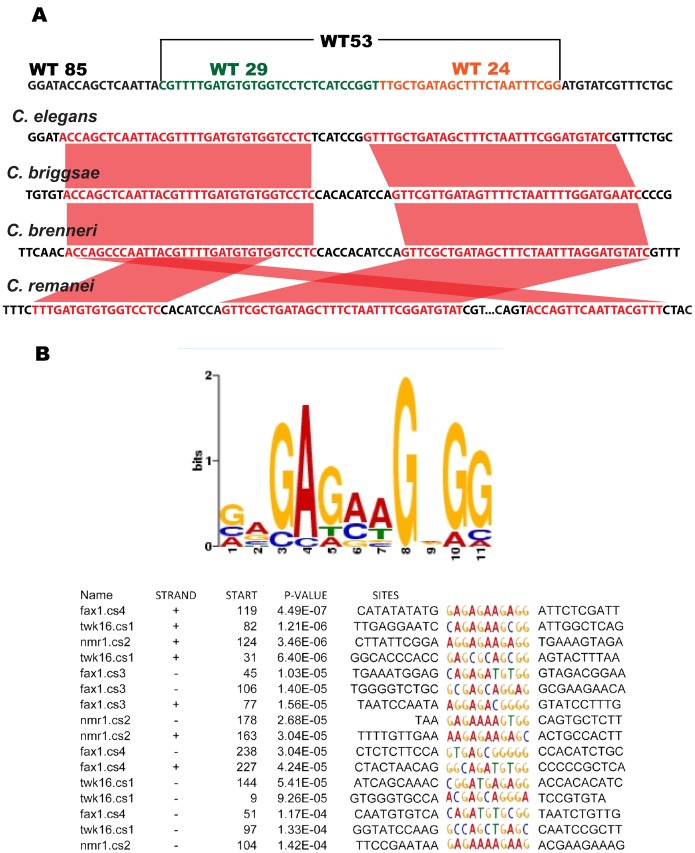
Sequence analyses. **A. Alignment of conserved** ***twk-16*** **sequences.** Sequence level comparison of the MUSSA alignment of the 73 bp conserved regions of twk-16.cs1 and orthologous *twk-16* regions from *C. elegans*, *C. briggsae*, *C. brenneri and C. remanei*. WT85 is shown at the top with the two sub-fragments of WT53 shown as WT29 in blue and WT24 in red type. WT53 is shown above in brackets. Conserved sequences in the four species MUSSA comparison using a window of 20 and threshold of 17 are in red type with red lines between the orthologs. **B. The consensus GA-rich motif identified by MEME.** The sequences representing the GA-rich motif in each fragment are highlighted in color type with flanking regions in black type. The strand is indicated as either+or – and start site of the GA-rich motif is indicated in each of the four genes used in the analysis. The respective genes and conserved regions used in the MEME analysis: fax-1.cs3 (180 bp), fax-1.cs4 (322 bp), nmr-1.cs2 (190 bp) and twk-16.cs1 (308 bp). The respective fragments contained 3 GA-rich motifs in fax-1.cs3, 4 GA-rich motifs in fax-1.cs4, 3 GA-rich motifs in nmr-1.cs2 and 3 GA-rich motifs in the 308 bp fragment containing twk-16.cs1. The 144 base start of the GA-rich motif in 308 bp twk-16.cs1 fragment corresponds to position 17 in the 53 bp WT53 and is shown in Figure 8B. The strand, start site, p-value and sequences were identified by MEME.

The 1.4 kb first intron of *twk-16* contains a region conserved between *C*. *elegans* and *C*. *briggsae* that drives expression in DVA [21]. A four-species, high stringency MUSSA analysis (20 bp window 17 of 20 bp identical) [8] identified twk-16.cs1 and twk-16.cs2 (Figure 2A). twk-16.cs1 contains a 73-bp conserved region (Figure 4A), which is contained in the region identified by Salkoff et al. [21]. The twk-16.cs2 region was 250 bp 3′ to twk-16.cs1 and contained four conserved subregions (twk-16.cs2.1, twk-16.cs2.2, twk-16.cs2.3 and twk-16.cs2.4) that were 259 bp in combined length.

### Identification of a Shared GA-rich Motif in *fax-1*, *nmr-1* and t*wk-16*

We compared the conserved, DVA-expressing sequences from *fax-1*, *nmr-1* and *twk-16* with MEME [26], seeking a shared single DVA consensus sequence. We included both a smaller 53-bp fragment WT53 of the *twk-16* intron, which did not produce restricted expression, as well as a larger 308-bp *twk-16* intronic fragment WT300 that restricted expression in the tail to DVA. A GA-rich motif was in all three conserved regions tested from *fax-1*, *nmr-1* and *twk-16*: four sites in WT300 containing twk-16.cs1, three sites of the 190 bp nmr-1.cs2 fragment and two sites of the 322 bp fragment fax-1.cs4 (Figure 4B). This motif spanned position 17–26 within the 53 bp of *twk-16* intron sequence present in WT53 (see Figure 8B).

In spite of the fact that this motif might arise from simple dinucleotide biases in the *C. elegans* genome [27], we tested its function in the context of the 190 bp nmr-1.cs2 element (nmr.WT190). DVA expression was significantly reduced when all GA-rich sites were mutated with transitions (C to T and G to A) (nmr.Mut190); Table 1; p<0.0001, Fisher’s Exact Test). However, the 100-bp fragment of the 190-bp nmr-1.cs2 that contains all three GA-rich motifs (nmr.WT100) failed to direct expression. Therefore, the GA-rich motif has a positive effect on gene expression but is not sufficient to drive expression. The opposite effect on expression was seen in the *twk-16* conserved regions in the WT53 element. Mutations across the GA-rich motif (Mut2) increased broad neuronal expression and ectopic expression in non-neuronal cells including the vulva. The GA-rich motif can repress transcription (p<0.0001; Table 1) in a context dependent manner in the *twk-16* enhancer.

**Table 1:** Expression of wild-type and mutated GA-rich motifs.

Line	Head	Tail	Ventral Cord	Pre-anal	Lumbar	DRG	DVA
nmr.WT190 (60)	100%	100%	73%	66%	97%	88%	88%
nmr.Mut190 (60)	78%	57%	50%	50%	48%	40%	40%
nmr.WT100 (30)	0%	3%	0%	0%	3%	0%	0%
WT53 (100)	84%	84%	53%	30%	75%	56%	47%
Mut2 (30)	100%	100%	100%	100%	100%	83%	83%

Summary of neuronal and DVA expression in transgenic *C. elegans* lines. Lines are labeled wild-type (WT) or mutated (Mut). The total number of animals scored is in parentheses with YFP expressing animals shown as a percentage of the total under the corresponding regions of the nervous system. The constructs used to generate the transgenic lines were: nmr.WT190 with conserved region nmr-1.cs2 and 3 WT GA-rich motifs; nmr.Mut190 with nmr-1.cs2 with mutations of 3 GA-rich motifs; nmr.WT100 with 100 bp of nmr-1.cs2 and 3 wild-type GA-rich motifs; WT53 with 53 bp of twk-16.cs1 and 1 wild-type GA-rich motif at position 17–27; and Mut2 with mutation of 8/10 residues of the 1 GA-rich motif in WT53.

### 85 bp of *twk-16* Intron is Sufficient for DVA Specific Expression

**Figure 5:**
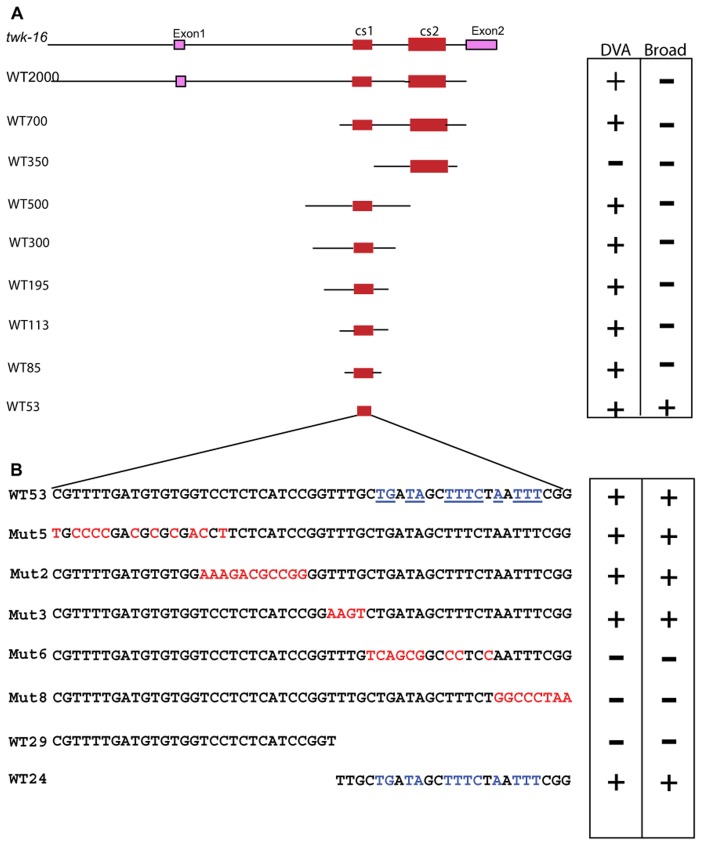
Mutation analysis. A. Analysis of the *twk-16* intron and enhancer. Deletion analysis of the *twk-16* intron with 73 bp twk-16.cs1 (cs1) and 259 bp twk-16.cs2 (cs2) denoted in red with flanking sequences in black and not to scale. The approximate sizes of the wild-type (WT) sequences are denoted by numbers from WT2000 to WT53. WT2000 was a plasmid construct and contains 500 bp 5′ of exon 1, exon 1 and 1.4 kb of the first intron containing both cs1 and cs2. WT700 contains 38 bp of flanking sequences 5′ to cs1 and 244 bp of flanking sequences 5′ to cs2 and 102 bp of 3′ flanking sequences. WT350 contains 55 bp of flanking sequences 5′ to cs2 and 45 bp of 3′ flanking sequences. WT500 contains 202 bp of flanking sequences 5′ to cs1 and 223 bp of 3′ flanking sequences. W300 contains 114 bp of flanking sequences 5′ to cs1 and 121 bp of 3′ flanking sequences. WT195 contains 114 bp of 5′ flanking sequence to cs1 and 8 bp of 3′ flanking sequence. WT113 contains 23 bp of 5′ flanking sequences to cs1 and 17 bp of 3′ flanking sequence. WT85 contains 4 bp of 5′ flanking sequences to cs1 and 8 bp of 3′ flanking sequence. WT85 contains WT53 with 17 bp of 5′ flanking sequence and 15 bp of 3′ flanking sequence. WT53 contains 53 bp of the 73 bp twk-16.cs1 region. **B.** **Mutational analysis of WT53.** The wild-type sequence is denoted by black type with the mutations of WT53 shown in red type. Conserved sequences identified by the seven species MUSSA comparison are in blue type with blue underlining. WT29 and WT24 are generated by cleavage of WT53 within the Mut3 region. DVA or broad neuronal expression is denoted by+or – in the box to the right of each construct.

**Figure 6:**
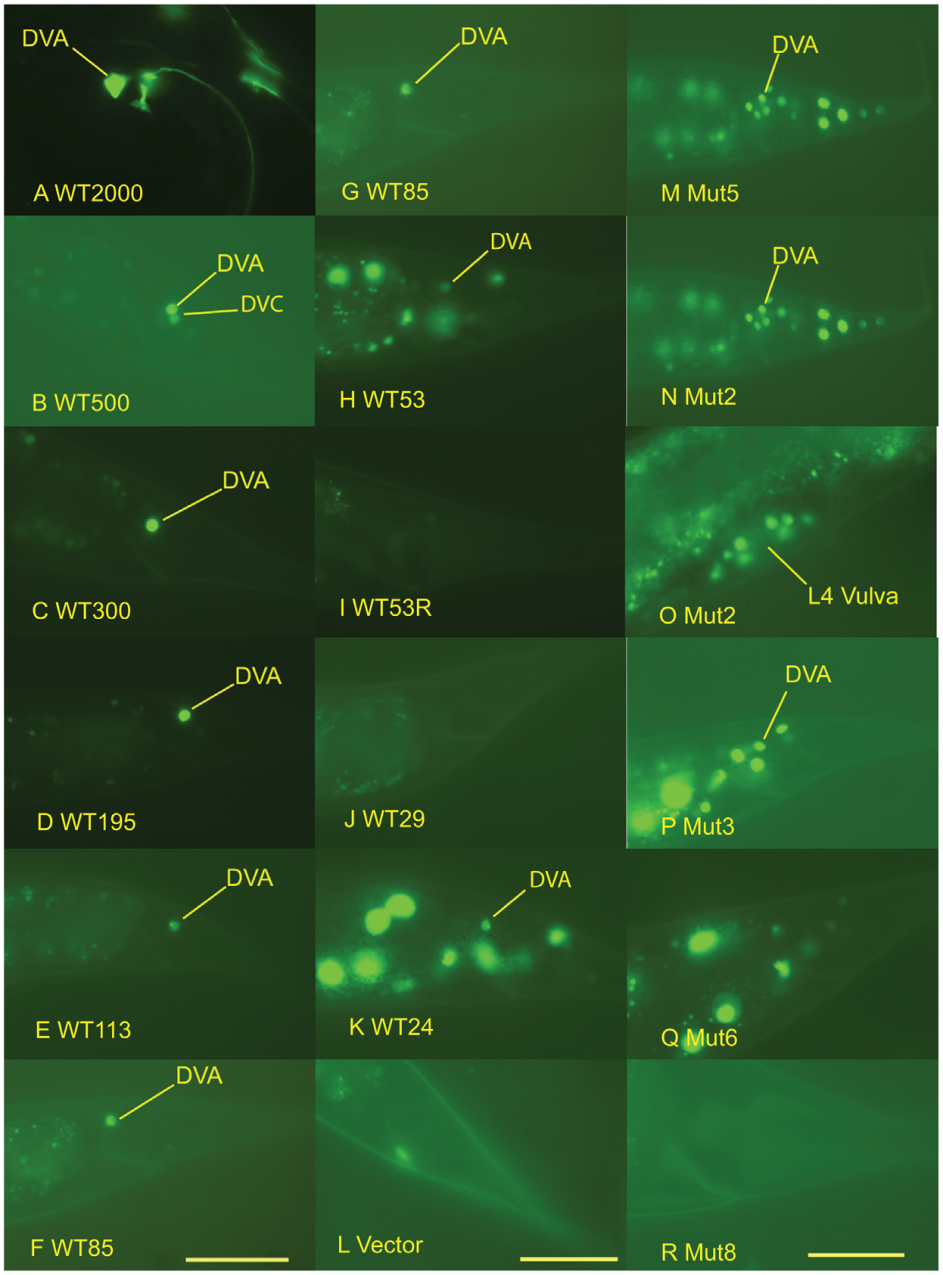
Photomicrographs of the expression of *twk-16* constructs in transgenic lines. The photomicrographs are arranged from left to right in three columns of six photomicrographs. **DVA expression of wild-type** ***twk-16*** **intron constructs.** Panels **A–F** are photomicrographs of the tail region of transgenic L4-adult *C. elegans* generated with the experimental sequences shown in Figure 5A. Yellow lines indicate DVA neurons expressing YFP. The constructs used to generate the transgenics in each panel were: **A**. WT2000∶500 bp of the 5′region of *twk-16* gene, the first exon and entire 1.4-kb first intron with twk-16.cs1 and twk-16.cs2. **B**. WT500: twk-16.cs1 and flanking sequence producing both DVA and DVC expression. **C**. WT300: twk-16.cs1 and flanking sequence **D**. WT195: twk-16.cs1 and flanking sequence. **E**. WT113: twk-16.cs1 and flanking sequence. **F**. WT85: twk-16.cs1 and short flanking sequences. **Expression of wild-type** ***twk-16*** **.cs1 constructs.** Panels **G–L** are photomicrographs of the tail of transgenic L4-adult *C. elegans* animals with the following constructs: **G**. WT85∶53 bp of twk-16.cs1 with 17 bp 5′ and 12 bp 3′ of flanking sequence. **H**. WT53∶53-bp fragment of twk-16.cs1 in wild-type orientation. **I**. WT53R in reverse orientation (3′-5′) to the expression vector. **J**. WT29: the 5′ 29 bp of WT53. **K**. WT24: the 3′ 24 bp of WT53 **L**. Vector: no experimental sequence and PCR expression vector Δ*pes-10*::4X NLS::YFP::*unc-54*::*unc-119*. **Expression of mutated** ***twk-16*** **.cs1 constructs.** Panels **M–R** are photomicrographs of the tail region of transgenic L4-adult *C. elegans* animals made with the following constructs containing mutations (Mut2-Mut8) of the 53-bp fragment (WT53) of the twk-16.cs1 region: **M**. Mut5. **N**. Mut2. **O**. Mut2. **P**. Mut3. **Q**. Mut6. **R**. Mut8. L4 Vulva expression in Mut2 transgenic is shown in Panel O with a yellow line identifying the vulva. Scale bars are specific to each column of six photomicrographs and = 20 µm.

Because of the highly restricted pattern of expression directed by constructs containing twk-16.cs1 but with additional flanking sequence, we analyzed this enhancer in more detail. In particular, we made constructs containing the regions identified by phylogenetic footprinting (Figure 4A) along with varying amounts of flanking sequences (Figure 5A). These constructs (except WT2000) used the Δ*pes-10* promoter (Figure 2B). WT2000 which contains 500 bp of sequence 5′ of the first exon, the first exon and the entire 1.4 kb of the first intron was used to produce transgenic lines [21]. WT2000 produced restricted GFP expression in the tail in DVA, along with one cell in the head tentatively identified as the amphid socket cell AMsoR. The expression seen in WT2000 represents expression of the *twk-16* enhancer with the 5′ wild-type regulatory regions; the expression is cytoplasmic because the GFP reporter lacks nuclear localization signals (Figure 6A). Constructs containing the first 2 kb of sequence 5′ to the first exon of *twk-16* without the first intron enhancer did not produce detectable expression.

A second conserved region (twk-16.cs2) is located 250 bp 3′ of the first conserved region in the first intron (Figure 2A). A construct containing both the twk-16.cs1and twk-16.cs2 regions of the first intron (WT700) produced the same expression pattern as constructs containing twk-16.cs1 but twk-16.cs2 alone (WT350) directed expression nowhere in the animal (Table 2). Therefore, only the twk-16.cs1 region possesses all of the elements necessary to produce restricted expression in DVA. This region can confer DVA expression at a distance, consistent with its prior characterization as an enhancer [21]. The WT500 construct sometimes expressed in both DVA and DVC (Figure 6B). Constructs of 308 bp (WT300) and 195 bp (WT195) produced the qualitatively brightest YFP expression in DVA (Figure 6C, 6D). The smallest fragment that could produce expression restricted to DVA was the 85 bp WT85 (Figure 6F, 6G). A 53 bp subfragment (WT53) produced qualitatively dimmer expression in DVA and broadly in other neurons (Figure 6H).

**Table 2:** Expression of wild-type *twk-16* intron constructs.

Line	Head	Tail	Ventral Cord	Pre-anal	Lumbar	DRG	DVA
WT2000 (30)	97%	97%	0%	0%	0%	93%	93%
WT700 (30)	67%	87%	0%	7%	20%	77%	63%
WT350 (20)	0%	0%	0%	0%	0%	0%	0%
WT500 (35)	100%	100%	0%	0%	0%	100%	100%
WT300 (17)	82%	100%	0%	0%	0%	100%	100%
WT195 (70)	70%F	100%	6%	3%	43% F	100%	100%
WT113 (60)	62%	65%	0%	0%	0%	65%	65%
WT85 (59)	0%	97%	0%	0%	0%	97%	97%
WT53 (100)	84%	84%	53%	30%	75%	56%	47%
WT53R (60)	67%	65%	8%	5%	60%	0%	0%
WT29 (30)	10%	20%	0%	0%	20%	0%	0%
WT24 (60)	100%	100%	100%	98%	100%	80%	78%

Summary of expression in transgenic *C. elegans* lines. Lines are denoted as wild type (WT) followed by a number with approximate size (bp) of the *twk-16* experimental sequence. The total number of animals scored is in parentheses with YFP expressing animals shown as a percentage of the total under the corresponding regions of the nervous system. All constructs (except plasmid WT2000) were made by PCR fusion with the expression vector shown in Figure 2B. The constructs used to generate the transgenic lines were: WT2000 with 500 bp 5′ of exon 1, exon 1 and the 1.4-kb first intron. The experimental sequences used were the following constructs (Figure 5A): WT700 with twk-16.cs1 and twk-16.cs2 regions and flanking sequence; WT350 with twk-16.cs2 region and flanking sequence; WT500 with twk-16 cs.1 region and flanking sequences; WT300 with twk-16.cs1 region and flanking sequence; WT195 with twk-16.cs1 region and flanking sequence; WT113 with twk-16.cs1 region and flanking sequence; WT85 with twk-16.cs1 and short flanking sequence; WT53 with 53 bp of twk-16.cs1; and WT53R is the reverse complement of WT53. The sub-fragments of WT53 are the 5′ 29b bp of WT53 (WT29) and the 3′ 24 bp of WT53 (WT24) (Figure 5B). The only individual neuron scored was DVA. (F signifies faint YFP expression).

This smaller 53 bp fragment of the twk-16.cs1 (WT53) showed YFP expression in DVA but also in other neurons in the tail, ventral cord and head (Table 2). WT53 also consistently expressed in the RID neuron in the head located in the dorsal pharyngeal ganglion. RID is not known to express *twk-16*, but is known to have direct reciprocal axonal connections to the DVA neuron. The conserved sequences in WT53 thus can direct expression in neurons both in the tail and elsewhere, but are not sufficient to restrict expression to DVA. When this same 53 bp of sequence (WT53) was placed in reverse orientation (WT53R) to the transcription cassette, there was dim expression in head and some tail neurons but no expression in DVA (Figure 6I, Table 2). Thus, WT53 may lack sequences conferring orientation-independence on the native *twk-16* enhancer.

### The Central 53 bp of the 85 bp WT85 Contains Positive and Negative Elements

To analyze the regions within WT53 responsible for DVA and broad neuronal expression we mutated nucleotides predicted by conservation and MEME to be required for expression in DVA (Figure 5B). Table 3 shows the percentage of animals expressing YFP in different regions of the nervous system in constructs in which sites identified computationally were mutated with transitions (C to T and G to A). Mutation of the predicted site (Mut5; Figure 5B) caused this element to direct broad expression in neurons in the head, ventral cord (VC), pre-anal ganglion (PA) and lumbar ganglion (LG) and DVA (Table 3; Figure 6M). Mutations within the first 29 bp (WT29) of WT53 promoted transcriptional activity, showing qualitatively brighter and broader patterns of expression than WT53. Mutant 2 (Mut2) increased the frequency of expression in neurons in head, VC, PA, LG and DVA compared to WT 53 (p<0.0001) (Table 3). There was also ectopic expression in intestinal cells and in the vulva (Figure 6N–O). Mutation 3 (Mut3) drove expression in the same pattern and frequency as WT53 (Figure 6P) except some Mut3 lines showed expression in hypodermal cells. The mutations in Mut5 and Mut2 in WT53 produced the most consistent neuronal expression (p<0.0001) (Table 3; Figure 6M–N), consistent with sequences in the Mut5 and Mut2 regions acting to repress expression.

**Table 3:** Expression of wild-type and mutated *twk-16* constructs.

Mut.	Head	Tail	Ventral Cord	Pre-anal	Lumbar	DRG	DVA
WT195 (70)	70%F	100%	6%	3%	43% F	100%	100%
Mut195 (60)	43%F	0%	0%	0%	0%	0%	0%
WT85 (59)	0%	97%	0%	0%	0%	97%	97%
Mut85 (60)	18%	21%	0%	0%	21%	0%	0%
5′ Mut85 (100)	0%	0%	0%	0%	0%	0%	0%
3′ Mut85 (100)	0%	0%	0%	0%	0%	0%	0%
5′3′Mut85 (100)	0%	0%	0%	0%	0%	0%	0%
WT53 (100)	84%	84%	53%	30%	75%	56%	47%
WT29 (30)	10%	20%	0%	0%	20%	0%	0%
WT24 (60)	100%	100%	100%	98%	100%	80%	78%
Mut5 (45)	100%	100%	100%	100%	100%	100%	100%
Mut2 (30)	100%	100%	100%	100%	100%	83%	83%
Mut3 (20)	100%	100%	90%	95%	95%	65%	50%
Mut6 (30)	83%	47%	27%	27%	47%	0%	0%
Mut8 (30)	3%	0%	0%	0%	0%	0%	0%

**Figure 7:**
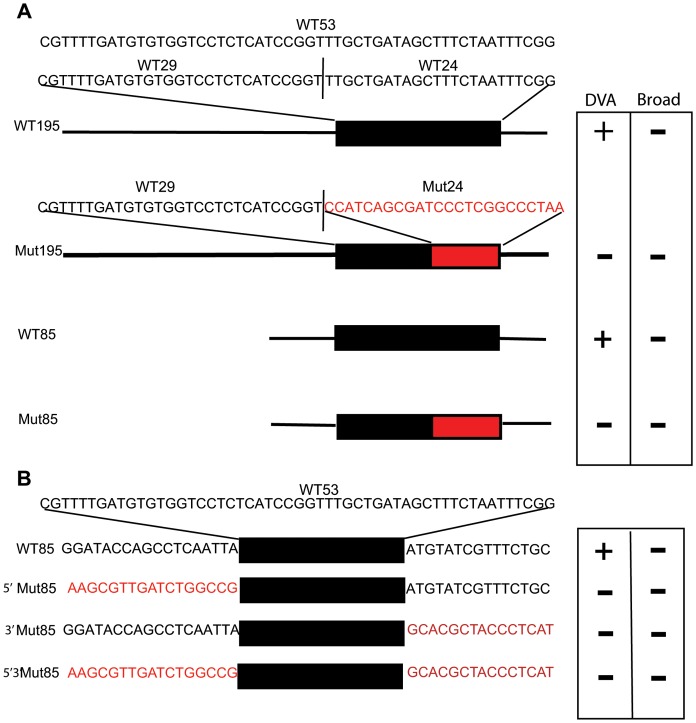
**A. Mutation of WT24 in the context of larger fragments.** The sequences of WT53 and sub-fragments WT29 and WT24 are shown in black type. WT53 is the black box in the diagrams of WT195 and WT85. Mutations of the bases of WT24 are in red type in Mut24. Mut24 is shown as a red box in the diagrams of Mut195 and Mut85. Neuronal expression in transgenic lines derived from the four experimental sequences is shown in the box as either+or – expression under DVA or Broad. **B.** **Mutation of the flanking sequences of WT53.** The WT53 sequence is shown in black type at top. WT53 sequence is represented by the black box with flanking wild-type sequences found in WT85 in black type. Mutations of the flanking sequences are shown in red type and labeled: 5′ Mut85 (mutation of the 17′bp 5′ of WT53); 3′ Mut85 (mutation of the 14 bp 3′ of WT53); and 5′3′ Mut85 (mutation of the 5′ and 3′ flanking sequences). Expression in DVA or Broad neuronal expression is denoted as+or - in the box to the right of the experimental sequences.

Summary of expression of wild-type and mutated *twk-16* constructs in transgenic *C. elegans* lines. The total number of animals scored is in parentheses with YFP expressing animals shown as a percentage of the total under the corresponding regions of the nervous system. Lines were produced with PCR fusion constructs with the expression vector shown in Figure 2B. Lines are denoted as wild-type (WT) or mutated (Mut) followed by the size (bp) of the experimental sequences. The experimental sequences in WT195 to 5′3′Mut85 are diagramed (Figure 7A, 7B) and were as follows: WT195 with WT24 sequences; Mut195 with mutations of WT24 sequences; WT85 with WT24 sequences; Mut85 with mutations of WT24; 5′Mut 85 with mutations of 5′ 17 bp of WT85; 3′Mut85 with mutations of 3′ 15 bp of WT85; and 5′3′Mut85 with mutations of both 5′ 17 bp and 3′ 15 bp of WT85. The experimental sequences used in constructs WT53 to Mut8 are shown in Figure 5B.

Since the Mut3 mutations did not change the frequency of DVA expression or the pattern of expression, we split WT53 into two fragments (the 5′ 29 bp, WT29, and 3′ 24 bp, WT24; Figure 5B). WT29 showed no expression in DVA and either no or barely detectable expression in other neurons (Table 2; Figure 6J). By contrast, WT24 drove expression in DVA, tail neurons, PA, VC and multiple head neurons, including RID (Table 2; Figure 6K). The pattern of expression was similar to that seen with the 53-bp fragment (WT53), but occurred in a higher percentage of animals (p<0.0001) and was qualitatively brighter (Figure 6K).

This 24 bp (WT24) region contains sequences required for both broad neuronal expression and DVA expression. Mutation 6 (Mut6) showed expression in the head neurons, but reduced expression in VC, PA and LG, and abolished expression in any of the DRG neurons including DVA (Table 3; Figure 6Q). Mutation 8 (Mut8) largely abolished expression in all neurons and cells in all lines, except for a few (3%) animals showing expression in head neurons (Table 3; Figure 6R). When the WT24 element was mutated in the context of larger fragments (Mut195 and Mut85) that showed highly restricted expression to DVA expression, all expression was abolished in DVA and all neurons (Figure 7A).

**Figure 8:**
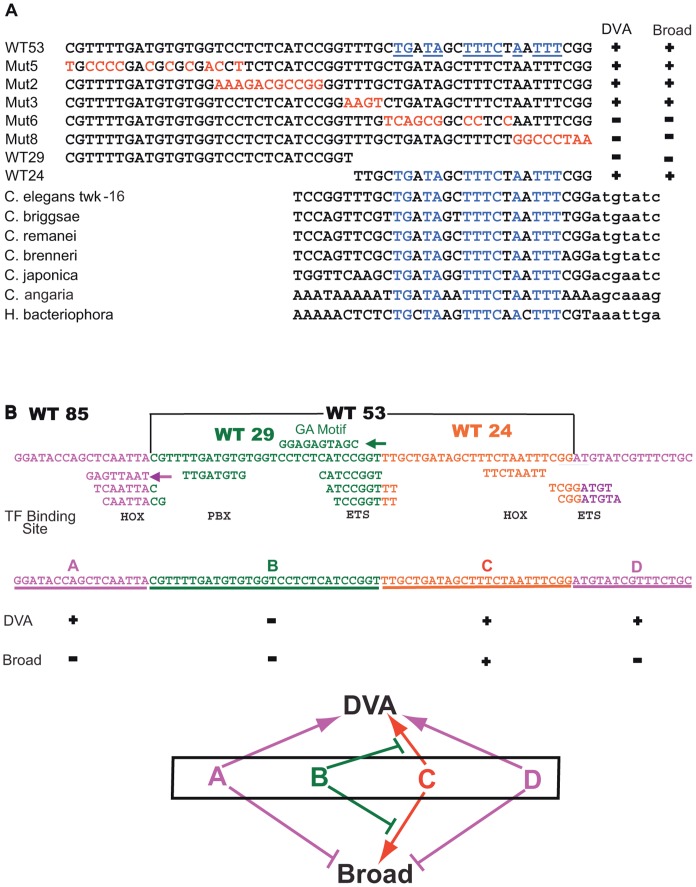
**A. Seven species comparison of** ***twk-16*** **enhancer and model.** The WT53 element is in black type and highly conserved bases identified by seven species MUSSA analysis in blue type and underlined. The experimental sequences of Mut2-Mut8 (Figure 5B) containing mutations of WT53 sequence are shown in red type and wild-type sequences of WT29 and WT24 in black type. Neuronal expression is denoted by+or – under DVA or Broad. MUSSA comparison of 3′-ward WT53 or WT53-like sequences from the *twk-16* genes or homolog’s of seven nematode species: *C. elegans*, *C. briggsae*, *C. remanei*, *C. brenneri*, *C. japonica*, *C. angaria* and *Heterorhabditis bacteriophora*. WT53 and WT53-like sequences are in uppercase; adjacent 3′-ward residues are in lowercase. Conserved bases shared by all seven species are in blue type. **B. Model of the 85 bp** ***twk-16*** **enhancer.** The WT85 sequence showing the A-D regions: A region 17 bp (purple); the D region 15 bp (purple); B region 29 bp (green); and C region 24 bp (orange). UniPROBE predicted TF binding sites for homeodomain TF’s (denoted by HOX) and ETS family TF’s (ETS) are shown below as colored sequence corresponding to the WT85 sequence. The GA-rich motif (green) in the B region with green arrow denoting the GA-rich motif is on the minus strand. Below the A-D regions is a summary of effects on neuronal expression as+or - of the four regions in rows labeled as DVA or Broad. The model diagram shows the A-D regions as letters with the same color scheme as the above WT85 sequence. Lines with arrowheads designate a positive effect on expression and the lines ending with a vertical line designate a negative effect on DVA or Broad neuronal expression.

### Short Flanking Sequences Restrict the Expression of WT53 to DVA

The initial phylogenetic comparison of the *twk-16* gene identified a 73 bp conserved region, which failed to drive expression, but that contains a 53 bp region that does drive expression in DVA and broad neuronal expression. Subsequent deletion analysis identified short regions (17 bp 5′ and 15 bp 3′) flanking the central 53 bp fragment that restricted expression to the DVA neuron. Specifically, mutation of either the 5′ 17 bp of the WT85 (5′Mut WT85) or 3′ 15 bp of WT85 (3′Mut WT85) abolished all expression in all lines examined (Figure 7B; Table 3). Expression was also abolished with mutations of both the 17 bp 5′ and 15 bp 3′ regions of WT85 (5′ 3′ Mut85; Figure 7B; Table 3). These flanking sequences are contained in the WT85 construct but were not identified by MUSSA using stringent parameters in the phylogenetic comparisons of four nematodes. The WT85 element produced consistent expression restricted to DVA with some lines showing faint expression in a few head neurons. These small flanking regions are thus required for expression in DVA and other neurons, and paradoxically, the restriction of expression to DVA.

### Seven-species Comparison Identifies 8 bp Required for Neuronal Expression

While WT53 is almost invariant in five *Caenorhabditis* species, a vast diversity of nematode species exist outside the Elegans group [28], [29], among which might exist versions of WT53 with recognizable but significant divergence from *C. elegans twk-16*. To test this idea, we identified *twk-16* orthologs in the newly sequenced genomes of *Caenorhabditis angaria* (PS1010; *Can-twk-16*); [30], *Pristionchus pacificus* (*Ppa-twk-16*; [29], and *Heterorhabditis bacteriophora* (*Hba-twk-16*; [31], [32] (X. Bai, B J Adams, TA Ciche, S Clifton, R Gaugler, K Kim, J Spieth, P W Sternberg, R K Wilson and P S Grewal, in preparation). We then searched their non-coding DNA with MUSSA for matches to the larger 308 bp intronic fragment WT300. In *Ppa-twk-16*, we found only one match in a minor intron to a functionally uncharacterized segment of *C. elegans* WT300. In contrast, the 5′ flanks of both *Can-twk-16* and *Hba-twk-16* each showed two strong matches to the ends of WT53. For the *twk-16* genes of seven nematode species, a single region of WT53 similarity showed consistent, transitive ungapped similarity (Figure 8A). These matches had the same orientation towards *twk-16* as in *C. elegans* and correlated strikingly with residues required for WT53 function in vivo (Mut8; Figure 6R). Mutation of these highly conserved bases in Mut8 of WT53 completely abolished expression in DVA and all other neurons and non-neuronal cells (Table 3; Figure 6R).

## Discussion

Some *C. elegans* neurons use neuron specific motifs to co-regulate neuron specific gene expression, as evidenced by analysis of AIY, ASE and AWB [9], [11], [14], [15], [33]. We tested whether expanded phylogenetic comparisons could reduce the experimental work required to identify regulatory regions and identify a shared cis-regulatory motif that resulted in the selective expression of genes in the DVA neuron. Phylogenetic comparisons of three or four nematode species did identify conserved regions at a comparable 66% (8/12) identification rate to the *ceh-13/lin-39* Hox locus (77%) [8]. In our analyses, there was a lower identification rate of 33% (4/12) for conserved regions that produced expression in DVA, consistent with the modular nature of regulatory regions and the evolutionary divergence of regulatory regions with increasing evolutionary distance [34]. However, phylogenetic footprinting of the *twk-16* genes from seven nematode species identified a highly conserved 8 bp that is necessary for expression in DVA and other neurons, suggesting that expanded phylogenetic comparisons are useful.

Our results also suggest limitations of phylogenetic comparisons. Even when using stringent parameters, 6 of 10 genes contained more than four conserved regions, a degree of conservation that does not substantially reduce the experimental work of testing regulatory regions. Expanding the number of species might help [34]. Highly conserved non-coding regions often have no positive effect on the particular aspect of transcription under study. A final shortcoming of phylogenetic comparisons is illustrated by the sequences responsible for restricted expression in DVA in the 5′ and 3′ ends of the 85 bp region. These sequences were not identified by our phylogenetic comparison because of the stringent parameters used in our initial comparison. Finding adjacent non-conserved regulatory sequences is consistent with our prior study of the *ceh-13*/*lin-39* Hox locus of *C*. *elegans*, where regulatory sequences were near, but not within, blocks of highly conserved DNA sequence [8]. This is consistent with the observation that the relative positions can be weakly conserved across species or diverge sufficiently to not be identified when using stringent parameters to reduce false positives [34].

### A Model for the *twk-16* Enhancer

The 85 bp *twk-16* DVA enhancer contains at least four regions with both positive and negative effects on gene expression (Figure 8B): a central core of 53 bp (WT53) containing the B (29 bp) and C (24 bp) regions; and two flanking regions, the A (17 bp) and D (15 bp) regions. The central C region is sufficient to drive both DVA and broader neuronal expression. Mutations in the 5′ B region when combined with the C region without the flanking A and D regions result in broad neuronal expression and ectopic expression in non-neuronal cells, consistent with the B region acting to repress expression. Removal of B from the central region results in broad and more robust expression consistent with B acting to repress the C region. While the central regions of B and C in combination are sufficient for DVA expression, it is not restricted to DVA. The addition of A and D to the central B and C regions restricts expression to DVA. Both the A and D flanking regions are also required for expression in DVA. However, mutation of the A and or D regions abolishes all neuronal expression while deletion of the A and D regions from the central B and C regions drives DVA and broad expression (Figure 7B). The model does not explain this discordance, suggesting that sequence specific and cell specific context dependence mediate the divergent effects of the A and D regions on neuronal expression. An alternative explanation for this discordance is that the A and D regions are required for expression in the context of the 85 bp element but not required in smaller fragments (WT53 and WT24) for both DVA and broad expression. In either case, the 85 bp element has multiple positive and negative acting sites that together can direct appropriate expression.

### Potential Transcription Factor Binding Sites in the *twk-16* Enhancer

We used the multi-species UniPROBE dataset of transcription factor binding sites [35] (http://the_brain.bwh.harvard.edu/UniPROBE) and cis-regulatory motifs archived in WormBase [22] to search for potential transcription factor binding sites within the *twk-16* enhancer. UniPROBE analysis predicted four potentially interesting binding sites in the 85 bp *twk-16* intronic region (Figure 8B; Figure S2). One predicted homeodomain binding site is within the 17 bp, positively acting A region. The negatively-acting B region has a predicted binding site for mouse Pbx-1, a homeodomain-containing transcription factor [36]. *C*. *elegans ceh-20,* is orthologous to the *Drosophila* HOX co-factor Extradenticle (Exd/Pxd), known to function as co-factor for homeodomain transcription factors [37]. *ceh-20* is expressed in many and possibly all neurons and thus could co-operatively repress broad neuronal expression of the *twk-16* enhancer. A binding site for an ETS family TF is predicted in region B directly overlapping the GA-rich motif. These sequences appear to repress the positive regulatory sequences in region C. A second homeodomain binding site is predicted in region C, necessary for expression in all neurons. This homeodomain binding site also overlaps the highly conserved 8 bp region identified by the phylogenetic comparison (Figure 8B). UniPROBE predicted many homeodomain transcription factor binding sites at this site (Figure S2), including vertebrate Alx3, Dlx2, Lhx2, Lbx2 and Hlxb9. The respective *C*. *elegans* orthologs include a group of homeodomain-containing transcription factors previously identified as being involved in the regulation of gene expression in the AIY neuron, including *ttx-3* and *ceh-10* [38]. The B and C regions in combination consistently drive expression in the RID neuron, and *ceh-10* is expressed in RID [39]. Additional *C*. *elegans* homeodomain transcription factors in this group include *ceh-14* and *lin-11*, both expressed in the lumbar ganglion. Consistent expression of WT53 is seen in the lumbar ganglion and this is attributed to the loss of flanking elements in regions A and D. There is a second predicted ETS binding site overlying the junction of the C and D region. Mutation of this site within the D region also abolishes all neuronal expression.

### Cis-regulation of the Terminal Gene Battery in DVA

The cis-regulatory mechanisms identified in the *C. elegans* neurons AIY, ASE and AWB support the model that single elements binding about two transcription factors regulate terminal gene batteries; this type of regulatory logic has been shown in multiple species [40]. Our finding of the relatively more complex structure of the *twk-16* DVA enhancer DVA gene expression is not consistent with this model. Of course this is only one gene of the likely multiple subsets of DVA expressed genes and the logic might depend on individual genes or batteries.

## Methods

### Strain Handling

*C*. *elegans* strains were handled and maintained following standard protocols and experiments were conducted at 20°C [41].

### Bioinformatics and Genome Comparisons

The genomic sequence of *Heterorhabditis bacteriophora* was generously provided before publication by the Genome Sequencing Center of Washington University (X. Bai et al., manuscript in preparation). All other genomic sequences, protein sequences, and genomic coordinates of *twk-16* orthologs were from the WS200 release of WormBase or from our published data (*C. angaria*) [30]. The coordinates of WT300 and WT53 elements in *Caenorhabditis* genomes (Table S2 Table S2) were determined by MUSSA comparisons to *C. elegans* followed by BlastN against reference genomes [42]. Motifs were predicted by MEME run on the UCSD web server (http://meme.ncbr.net; [26]; ungapped blocks of similarity were detected by MUSSA run locally [8]. To exclude them from MUSSA comparisons to WT300 or WT53, exons of *twk-16* orthologs (or their neighbors, where applicable) were masked as ‘N’ residues with Perl.

Two contigs encoding the 5′- and 3′-ward halves of *Hba-twk-16* were detected by TBlastN with *C. elegans* TWK-16 against the *H. bacteriophora* genome assembly. Their sequences were oriented to have a consistent 5′-to-3′ direction for *Hba-twk-16*, and joined with a nominal 100 undetermined (‘N’) residues. *hog-1* and *pccb-1* homologs on the 5′ and 3′ sides of *Hba-twk-16* were found by BlastX against wormpep200; gene models for *Hba-twk-16, Hba-hog-1, and Hba- pccb-1* were predicted with *exonerate* and *C. elegans* protein sequences (arguments, “*-E -m protein2genome:bestfit*”; [43]).

MUSSA [8] was used to identify evolutionarily conserved sequences. MUSSA uses N-way transitivity (all-against-all) so that only windows passing the selected similarity threshold across all species are reported as alignments. The MEME Web server was used to identify nonaligned motifs shared by different sequences [44]. Possible instances of WormBase motifs in WT53 were detected with FIMO [45].

### Transgene Design and Construction

Unless otherwise noted, the transcriptional reporter gene constructs contained the test sequence 5′ to the minimal Δ*pes-10* promoter in Fire laboratory vector pPD122.53 [46] modified to contain YFP rather than GFP. These constructs were then fused by PCR to a second PCR construct [47] derived from pDPMM051, containing the 5′ non-coding region and an *unc-119* minigene [48]. The final construct utilized for ballistics was: *Experimental Sequence*:: Δ*pes-10*::4X NLS::YFP::*unc-54*:: *unc-119* (Figure 2B). Wild-type (WT) constructs ≤73 bp in size were synthesized as oligonucleotides and ligated to the vector. We mutated conserved sequences by synthesis with the substituted bases at the designated sites along with the 5′ Δ*pes-10* anchor sequence. Mutated sequences produced by oligonucleotide annealing and PCR fusion were sequenced to determine if the correct product was produced. The mutations of the GA-rich motif were produced by PCR fusion of a mini-gene (Integrated DNA Technologies, Coralville IA; IDT) derived PCR product to the above reporter vector. The minigenes were sequenced by the supplier (IDT).

### Transgenesis

*C*. *elegans* strain PS3460 [*unc-119(ed4)*] was transformed with transgenic constructs by micro-particle bombardment using the PDS-1000/He Biolistic system (Bio-Rad). For a detailed protocol contact the authors. Briefly, nematodes were grown with HB101 in S-complete synchronously in liquid culture from bleached eggs for 72 hours at 20 degrees to L4-early adult stages. They were then used for ballistics and were recovered in liquid culture for two days. Following the two-day recovery period, worms were concentrated and rimmed onto 10 cm plates with OP50 lawns to identify non-Uncoordinated transgenic larvae by their ability to emerge and crawl onto the bacterial lawn. Independent transgenic lines were maintained and examined for each reporter construct.

### Scoring of Transgenic Animals

Expression was scored by Nomarski optics and YFP expression on a Zeiss Axioplan microscope. Photographs were taken with a digital camera at 100x using Improvision Openlab software. Lines to be scored were selected by high frequency transmission of the non-Uncoordinated phenotype, and the presence of either visible expression by low power epifluorescence microscopy or no expression in all lines. Scoring was limited to regions, i.e. head or tail or regions or anatomically defined regions such as ventral cord (VC), pre-anal ganglion (PA), lumbar ganglion (L) or the dorsal rectal ganglion (DRG). The only neuron individually scored was DVA. Most transgenic lines produced by bombardment show a consistent pattern of expression between animals within each independent line. However, there were significant differences in YFP brightness qualitatively between lines produced with the same construct. We scored from three to ten animals from each independent line for each construct and scored from two to ten lines with an average of three lines (Table S1). In a few cases only two lines were generated but included in the data if attempts to get additional lines was unproductive. Expression in DRG was scored as positive if we saw expression in DVA or if we could see expression in DRG but not definitely identify the cell as DVA or the other two cells in the DRG, DVB or DVC. Statistical analysis of differences in the frequency of expression in DVA was performed when there were not clear differences between lines. Expression in DVA for different constructs was compared by Fisher’s Exact Test.

## Supporting Information

Figure S1 **Phylogenetic comparisons by MUSSA of four DVA expressed genes. A. MUSSA analysis of** ***acr-15*** **gene. B. MUSSA analysis of** ***fax-1*** **gene. C. MUSSA analysis of** ***nmr-1*** **gene. D. MUSSA analysis of** ***twk-16*** **gene.** Respective genes are shown above the regions analyzed by MUSSA with exons in either blue or pink with non- coding regions as black lines. The analyses included the 5′ intergenic regions of *C*. *elegans* genes *acr-15* (1.6 kb), *fax-1* (5.7 kb), *nmr-1* (1.1 kb) and *twk-16* first intron (1.4 kb). These non-coding regions were compared to the corresponding orthologous genes of *C*. *briggsae* (CBG) and *C*. *remanei* (CR) using a window of 20 and threshold of 17 ungapped identities (85% match) and shown as red lines between the orthologs.
(TIF)

Figure S2 **Uniprobe analysis of WT85.** WT85 was analyzed against all species TF’s in the Uniprobe database http://the_brain.bwh.harvard.edu/uniprobe). Predicted Homeodomain transcription factor binding sites (Homeodomain TF’s) and ETS family transcription factors binding sites (ETS Domain TF’s) are above the predicted binding sites for the TF’s represented by multiple colored lines, which correspond to the TF’s listed in the column.
(TIF)

Table S2 **Summary of transgenic lines.** The lines are listed by alphabetical name followed by number of that specific line, date scored, number of animals scored and neuronal expressio by region or ganglion. The only neuron scored individually was DVA. Scoring of animals was done as in Table.
(XLSX)

Table S2 **Genomic sequence coordinates of known or inferred regulatory elements.** Genomic coordinates in *C. elegans* are given for both the WS190 and WS215 releases of WormBase. All other genomes have coordinates from the WS190 release.
(DOCX)
