# Supplementary figures and images for: Identification of DVA Interneuron Regulatory Sequences in Caenorhabditis elegans
Source: PLoS One. 2013 Jan 28;8(1):e54971. doi: 10.1371/journal.pone.0054971 (PMC3557239; doi:10.1371/journal.pone.0054971)

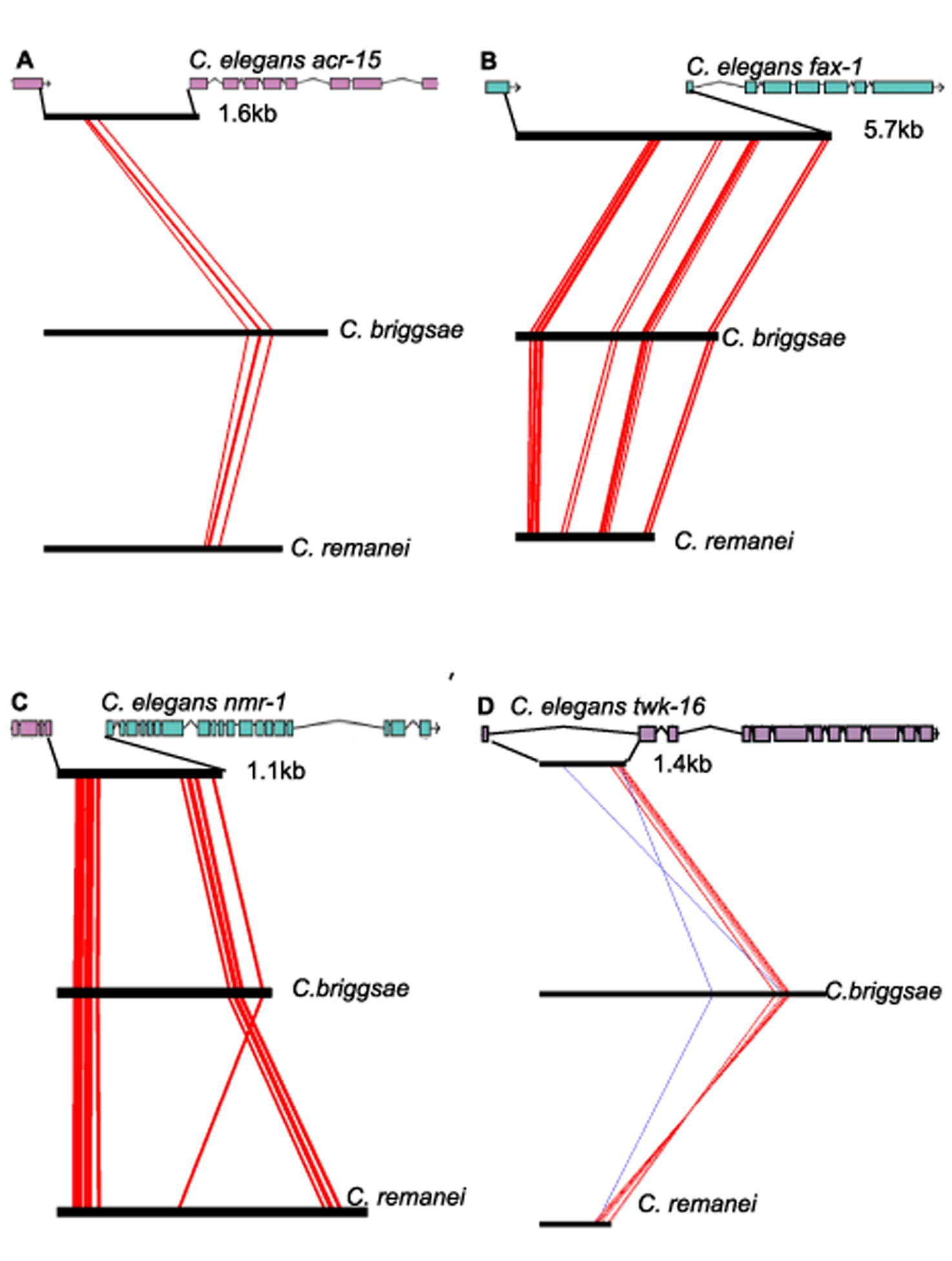

Supplement: Figure S1 — Phylogenetic comparisons by MUSSA of four DVA expressed genes. A. MUSSA analysis of acr-15 gene. B. MUSSA analysis of fax-1 gene. C. MUSSA analysis of nmr-1 gene. D. MUSSA analysis of twk-16 gene. Respective genes are shown above the regions analyzed by MUSSA with exons in either blue or pink with non- coding regions as black lines. The analyses included the 5′ intergenic regions of C. elegans genes acr-15 (1.6 kb), fax-1 (5.7 kb), nmr-1 (1.1 kb) and twk-16 first intron (1.4 kb). These non-coding regions were compared to the corresponding orthologous genes of C. briggsae (CBG) and C. remanei (CR) using a window of 20 and threshold of 17 ungapped identities (85% match) and shown as red lines between the orthologs. (TIF) [file pone.0054971.s001.tif]

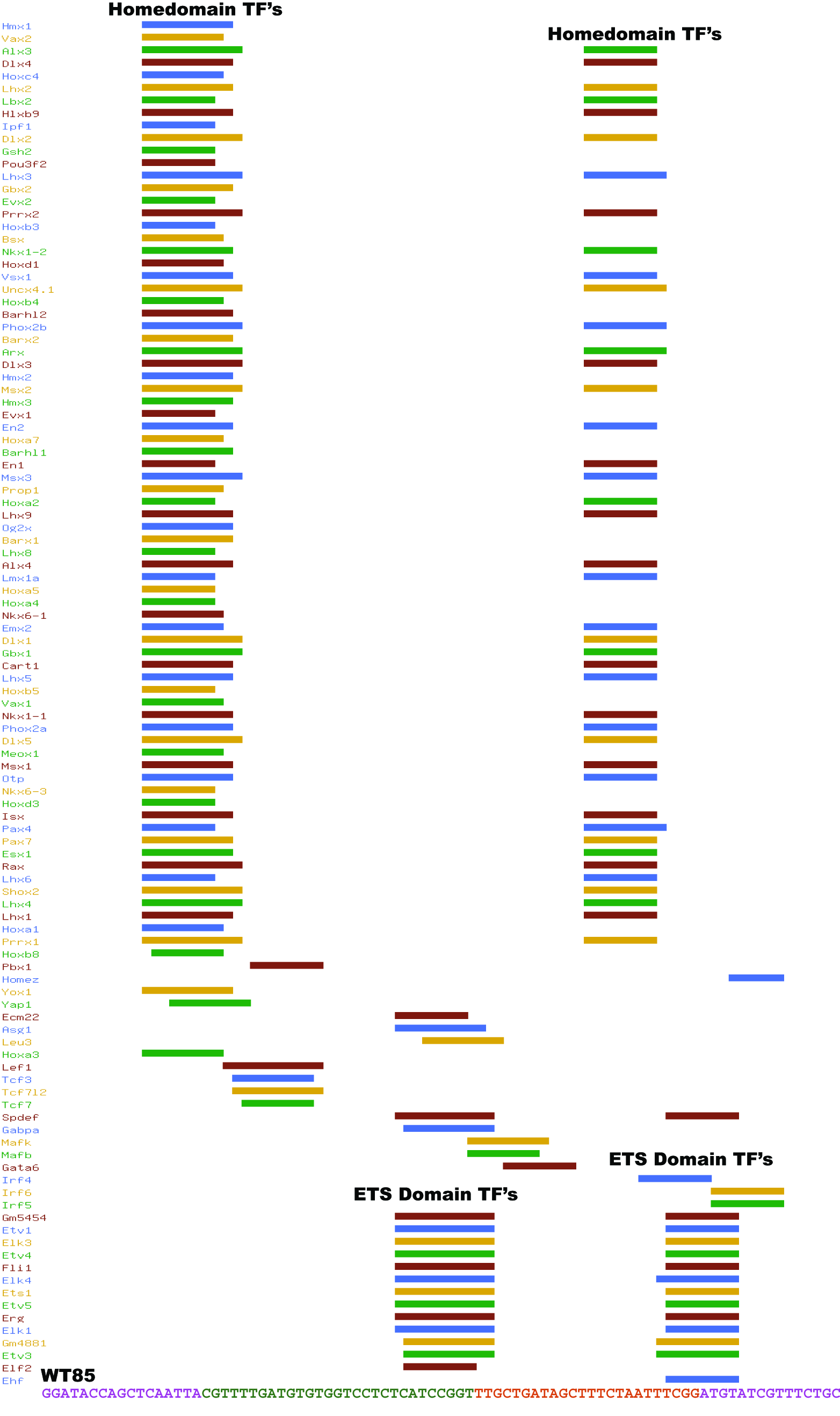

Supplement: Figure S2 — Uniprobe analysis of WT85. WT85 was analyzed against all species TF’s in the Uniprobe database http://the_brain.bwh.harvard.edu/uniprobe). Predicted Homeodomain transcription factor binding sites (Homeodomain TF’s) and ETS family transcription factors binding sites (ETS Domain TF’s) are above the predicted binding sites for the TF’s represented by multiple colored lines, which correspond to the TF’s listed in the column. (TIF) [file pone.0054971.s002.tif]
