# Supplementary material for: Identification of DVA Interneuron Regulatory Sequences in Caenorhabditis elegans
Source: PLoS One. 2013 Jan 28;8(1):e54971. doi: 10.1371/journal.pone.0054971 (PMC3557239; doi:10.1371/journal.pone.0054971)
Supplement: Table S2 — Genomic sequence coordinates of known or inferred regulatory elements. Genomic coordinates in C. elegans are given for both the WS190 and WS215 releases of WormBase. All other genomes have coordinates from the WS190 release. (DOCX) [file pone.0054971.s004.docx]

**Table S2**. **Genomic sequence coordinates of known or inferred regulatory elements.**

| **Element** | **Species** | **Genomic coordinates** | **Size/orientation** |
| --- | --- | --- | --- |
| WT300 | *elegans* | X:3082057..3082364 [WS215] | 308 bp |
|  |  | X:3082059..3082366 [WS190] |  |
|  | *briggsae* | chrX:7967486..7967804 | 319 bp |
|  | *remanei* | Crem_Contig0:4235360..4235678 | 319 bp; antisense |
|  | *brenneri* | Cbre_Contig0:1439376..1439700 | 325 bp |
|  | *japonica* | Cjap_Contig50:198652..198953 | 302 bp; antisense |
|  |  |  |  |
| WT53 | *elegans* | X:3082184..3082236 [WS215] | 53 bp |
|  |  | X:3082186..3082238 [WS190] |  |
|  | *briggsae* | chrX:7967486..7967804 | 319 bp |
|  | *remanei* | Crem_Contig0:4235360..4235678 | 319 bp; antisense |
|  | *brenneri* | Cbre_Contig0:1439376..1439700 | 325 bp |
|  | *japonica* | Cjap_Contig50:198652..198953 | 302 bp; antisense |

Genomic coordinates in *C. elegans* are given for both the WS190 and WS215 releases of WormBase. All other genomes have coordinates from the WS190 release.
